# Potential role of levocarnitine supplementation for the treatment of chemotherapy-induced fatigue in non-anaemic cancer patients

**DOI:** 10.1038/sj.bjc.6600413

**Published:** 2002-06-17

**Authors:** F Graziano, R Bisonni, V Catalano, R Silva, S Rovidati, E Mencarini, B Ferraro, F Canestrari, A M Baldelli, A De Gaetano, P Giordani, E Testa, V Lai

**Affiliations:** Medical Oncology Unit, Hospital of Urbino, Via Bonconte da Montefeltro, 61029 Urbino, Italy; Medical Oncology Unit, Hospital of Fabriano, Italy; Division of Medical Oncology, Hospital of Pesaro, Italy; ‘G. Fornaini’ Institute of Biological Chemistry, University of Urbino, Italy; Laboratory of Biostatistics, Centro Nazionale delle Ricerche (CNR), Rome, Italy

**Keywords:** fatigue, chemotherapy, cancer, carnitine

## Abstract

Ifosfamide and cisplatin cause urinary loss of carnitine, which is a fundamental molecule for energy production in mammalian cells. We investigated whether restoration of the carnitine pool might improve chemotherapy-induced fatigue in non-anaemic cancer patients. Consecutive patients with low plasma carnitine levels who experienced fatigue during chemotherapy were considered eligible for study entry. Patients were excluded if they had anaemia or other conditions thought to be causing asthenia. Fatigue was assessed by the Functional Assessment of Cancer Therapy-Fatigue quality of life questionnaire. Treatment consisted of oral levocarnitine 4 g daily, for 7 days. Fifty patients were enrolled; chemotherapy was cisplatin-based in 44 patients and ifosfamide-based in six patients. In the whole group, baseline mean Functional Assessment of Cancer Therapy-Fatigue score was 19.7 (±6.4; standard deviation) and the mean plasma carnitine value was 20.9 μM (±6.8; standard deviation). After 1 week, fatigue ameliorated in 45 patients and the mean Functional Assessment of Cancer Therapy-Fatigue score was 34.9 (±5.4; standard deviation) (*P*<.001). All patients achieved normal plasma carnitine levels. Patients maintained the improved Functional Assessment of Cancer Therapy-Fatigue score until the next cycle of chemotherapy. In selected patients, levocarnitine supplementation may be effective in alleviating chemotherapy-induced fatigue. This compound deserves further investigations in a randomised, placebo-controlled study.

*British Journal of Cancer* (2002) **86**, 1854–1857. doi:10.1038/sj.bjc.6600413 www.bjcancer.com

© 2002 Cancer Research UK

## 

In cancer patients, increasing attention is being devoted to the assessment and management of fatigue (Richardson and Ream, 1996; Stone *et al*, 2000). Fatigue is a common symptom in patients with advanced disease, and it is frequently reported as a side-effect of chemotherapy (Richardson and Ream, 1996; Morrow *et al*, 1999; Stone *et al*, 2000). Cancer-related fatigue causes a dramatic impairment of the physical status, but it is often underestimated and poorly managed by physicians (Stone *et al*, 2000). Physiological factors (anaemia, metabolic abnormalities, malnourishment), psychological factors (depression, sleep deprivation) or tumour-related factors (altered metabolic functions, cytokines) are among the known etiologies that contribute to fatigue (Portenoy and Itri, 1999). Many patients report fatigue a few days after their first chemotherapy treatment (Richardson and Ream, 1996; Morrow *et al*, 1999), but this early presentation is often unexplained by common predisposing factors or other evident underlying conditions (Portenoy and Itri, 1999; Schwartz *et al*, 2000).

In light of recent biochemical investigations, it is possible that carnitine deficiency may have a role in the development of chemotherapy-induced fatigue (Dodson *et al*, 1989; Breitkreutz *et al*, 2000; Peluso *et al*, 2000). In humans, carnitine derives from food intake or biosynthesis from the metabolism of lysine and methionine. Carnitine is produced in liver and kidneys, stored in skeletal muscle, and excreted mainly in urine. In mammalian cells, carnitine and its products play a central role in the energy metabolism. Carnitine is indispensable for glucose and lipid turnover, and it is essential for mitochondrial fatty acid oxidation, which is the primary fuel source in heart and skeletal muscle (Peluso *et al*, 2000). Chemotherapy causes dysfunction of the carnitine system, which may contribute to a condition of asthenia due to the impaired energy metabolism (Breitkreutz *et al*, 2000; Peluso *et al*, 2000). Ifosfamide and cisplatin alter major enzymatic pathways and they cause carnitine secondary deficiency after increasing urinary excretion of the molecule (Dodson *et al*, 1989; Heuberger *et al*, 1998; Marthaler *et al*, 1999).

Levocarnitine (LC) supplementation was found to restore normal plasma carnitine levels and resolve symptomatic deficiencies with excellent tolerability to treatment (Baker *et al*, 1993). In haemodialysis patients, LC treatment increased plasma carnitine concentrations and improved patient-assessed fatigue (Brass *et al*, 2001).

On this basis, we investigated whether LC supplementation may have a role in ameliorating chemotherapy-induced fatigue in non-anaemic patients with solid tumours.

## MATERIALS AND METHODS

### Patients

Consecutive patients with solid tumours who experienced fatigue during first-line, palliative chemotherapy containing ifosfamide or cisplatin were considered eligible for study entry. Inclusion criteria consisted of: ECOG performance status (PS) 0–1; normal renal and liver functions; adequate bone marrow reserve with haemoglobin (Hb) level ⩾13 g/dl; low plasma level of free carnitine (<30 μM). Also, adequate dietary/caloric intake (30–40 Kcal Kg day^−1^) and normal Body Mass Index (19–24 Kg h^2^) were evaluated by dietician consultants before study entry. Patients receiving corticosteroids, psychostimulants or vitamins and patients with anaemia or other underlying conditions associated with asthenia were excluded. The protocol was approved by each local institutional review board and all patients gave written informed consent.

### Fatigue assessment

Assessments of fatigue were performed using the Italian version of the Functional Assessment of Cancer Therapy-Fatigue (FACT-F) quality of life questionnaire (Yellen *et al*, 1997). This 13-item scale is a tool for the *ad hoc* measurement of fatigue and its influence on global quality of life of cancer patients. Each question is scored on a five-point scale rating from 0 (not at all) to 4 (very much). The FACT-F investigates fatigue symptoms over a 7-day period and lower FACT-F scores are associated with higher levels of fatigue. The FACIT scales are designed for patient self-administration or interview format. In this study, an investigator was trained at each Institution so as to elicit non-biased patient responses. During the interviews, patients held a card on which the response options were printed.

### Treatment plan with LC

According to the current knowledge of the carnitine system, its metabolism and treatment of deficiency, a high daily fractioned dose of LC for 7 days was chosen for this study. The dosage was spaced throughout the day (every 12 h), preferably during or following meals. It was found that daily administrations of 2–3 g of LC led to blood carnitine concentrations superior to normal levels (Bach *et al*, 1983; Baker *et al*, 1993; Brass *et al*, 2001). Rapid recovery of symptomatic plasma carnitine deficiency is obtained within 10 days by either oral high-dose treatment (3–4 g) in a single administration or by more prolonged therapy (Bizzi *et al*, 1978; Campos *et al*, 1993). Mucosal absoption of LC is saturated by 2 g doses and oral fractioned treatment for higher doses is required (Farper *et al*, 1988).

### Study design

Enrolled patients completed the baseline FACT-F questionnaire and supplied blood samples to determine their plasma levels of free carnitine (Deufel, 1990; Xia and Folkers, 1991). In the general population this value is reported as >30 μM (Deufel, 1990; Xia and Folkers, 1991) and in a preliminary analysis in 30 healthy subjects we found a mean value of 40.5 μM (s.d.; 33–48). After baseline evaluation, patients started treatment with oral LC 2 g solution B.I.D., for 7 days (Sigma Tau S.p.A., Rome, Italy). During treatment, patients were monitored weekly with physical examination, medical history, blood chemistries and FACT-F assessments until the next cycle of chemotherapy. Tumour response was evaluated according to the World Health Organisation criteria (WHO, 1979).

In this study, values were reported as means±standard deviation (s.d.). Differences in means between two or more groups were analysed using two-tailed Student's *t*-test or one-way ANOVA (Instat 3.0. GraphPad Software Inc, San Diego CA, USA). Values of *P*<0.05 were considered statistically significant.

## RESULTS

Between December 1999 and March 2001, 50 patients were enrolled in this prospective study. They were fully evaluable for the outcome analysis of LC supplementation and their characteristics are reported in Table 1

**Table 1:**
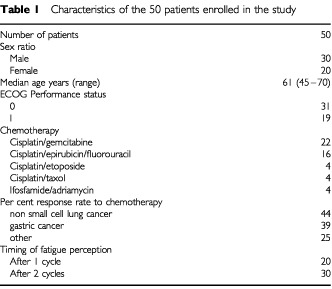
Characteristics of the 50 patients enrolled in the study

. All patients received combination chemotherapy for palliative treatment of stage IV solid tumours. It was cisplatin-based in 20 patients with non-small cell lung cancer, 16 patients with gastric cancer, four patients with small-cell lung cancer, four patients with ovarian cancer and two patients with pancreatic cancer. It was ifosfamide-based in four patients with soft-tissue sarcomas. Fatigue was complained of after the first cycle of chemotherapy in 20 patients and after the second cycle in 30 patients. In the 50 patients, baseline mean Hb level was 13.6 g dl^−1^ (±0.5; s.d.), mean FACT-F score was 19.7 (±6.4; s.d.) and the mean plasma carnitine value was 20.9 μM (±6.8; s.d.).

All the 50 patients received the planned treatment without LC dose reductions and each patient completed three follow-up FACT-F questionnaires. After 1 week, 50 patients showed post-treatment plasma levels of free carnitine >30 μM and fatigue ameliorated in 45 patients (90%). In Table 2

**Table 2:**
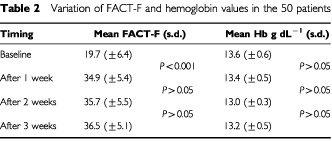
Variation of FACT-F and hemoglobin values in the 50 patients

mean FACT-F scores and mean haemoglobin levels at baseline (before starting LC) and subsequent weekly assessments are shown. The mean FACT-F scores after 1, 2 and 3 weeks following the baseline evaluation were: 34.9 (±5.4; s.d.), 35.7 (±5.5; s.d.) and 36.5 (±5.1; s.d.), respectively. The difference in FACT-F mean values between the baseline and the first post-treatment assessment was statistically significant (*P*<.001). Differences in post-treatment FACT-F assessments between the first and the second week, and between the second and the third week were not statistically significant (*P*>0.05). In the five non-responder patients, FACT-F scores remained stable in three patients and worsened in two patients.

## DISCUSSION

In mammalian cells, the carnitine system is essential for glucose and lipid turnover and it plays a crucial role in maintaining the energy metabolism (Breitkreutz *et al*, 2000; Peluso *et al*, 2000). Chemotherapy causes dysfunction of enzymes involved in the transport and trafficking of carnitines (Peluso *et al*, 2000) and, in addition, ifosfamide and cisplatin induce increased urinary excretion of these molecules (Dodson *et al*, 1989; Marthaler *et al*, 1999; Heuberger *et al*, 1998). These effects may worsen the dysmetabolic syndrome associated with cancer (Tisdale, 1997) and increase side-effects of chemotherapy, like fatigue (Tisdale, 1997; Portenoy and Itri, 1999).

Fatigue is a common symptom in patients with advanced cancer and it frequently occurs after anti-cancer therapies (Stone *et al*, 2000). In patients with metastatic tumours, cancer-related fatigue is extremely prevalent and it is observed in 70 to 80% of patients receiving chemotherapy and up to 90% of radiotherapy patients (Richardson and Ream, 1996; Morrow *et al*, 1999; Schwartz *et al*, 2000). On the basis of current experience, fatigue is considered primarily treatment-related when there is a clear relationship between the timing of fatigue and the therapeutic intervention. In these cases, chemotherapy-induced fatigue is an early side-effect which peaks within a few days after treatment and declines thereafter (Morrow *et al*, 1999). Cancer-related fatigue and chemotherapy-related fatigue are multifactorial, however, anaemia seems to play a major role (Portenoy and Itri, 1999). Recent experiences have demonstrated the relationship between mild-moderate anaemia, fatigue and quality of life (Groopman and Itri, 1999). Treatment of anaemia with epoetin alpha resulted in significant improvements of energy levels, activity levels, functional status and overall quality of life (Groopman and Itri, 1999). Current data suggest that patients with haemoglobin levels >12 gr dl^−1^ show significantly less fatigue and better quality of life than patients with haemoglobin values less than 12 gr dl^−1^ (Cleeland *et al*, 1999). Epoetins are the mainstay of treatment for anaemia-associated fatigue, but other conditions and mechanisms may sustain fatigue and require a different approach to treatment (Portenoy and Itri, 1999; Tisdale, 1997). Exercise and education about fatigue may be beneficial in its relief (Dimeo *et al*, 1999). Low-dose corticosteroids have shown some positive effects against fatigue but data remain scattered and unconfirmed in comparative trials (Portenoy and Itri, 1999). It was postulated that antidepressant modulating serotonin may alleviate fatigue in patients treated with chemotherapy. Unfortunately, the serotonin re-uptake inhibitor paroxetine was unable to improve fatigue in a double-blind, placebo-controlled trial (Morrow *et al*, 2001). Tumour-induced cytokines and host-produced pro-inflammatory cytokines may represent a possible mechanism contributing to a condition of fatigue. Current research is exploring these and other mechanisms which could be the target of future clinical trials for the treatment of fatigue (McNeil, 2001).

In the present study, a selected population of non-anaemic patients, with good performance status and without significant comorbidities showed early fatigue after cisplatin or ifosfamide-based chemotherapy. In the majority of patients, this side-effect significantly improved after LC supplementation which was well tolerated and did not affect anti-cancer therapeutic efficacy. These data suggest that chemotherapy-induced damage of the carnitine system and secondary deficiency of the molecule (Dodson *et al*, 1989; Heuberger *et al*, 1998; Marthaler *et al*, 1999; Peluso *et al*, 2000) may cause fatigue due to impaired energy metabolism (Peluso *et al*, 2000). It therefore follows that restoration of the carnitine pool may alleviate this symptom (Brass *et al*, 2001). To the best of our knowledge, this is the first study which has explored the therapeutic intervention of the carnitine system for treating selected patients with chemotherapy-induced fatigue. Results are encouraging, but these data should be looked at with caution due to potential biases and limitations inherent to the study itself.

In non-anaemic cancer patients, carnitine deficiency may not be the primary cause of fatigue and concomitant disease-related or host-related conditions may contribute to this toxicity. Early chemotherapy-induced asthenia may improve spontaneously and independently from specific interventions (Richardson and Ream, 1996; Morrow *et al*, 1999). Also, reduction of tumour burden in response to the anticancer treatment may alleviate fatigue. The urinary excretion of carnitine persists for several days after chemotherapy and this clearance returns to normal values 7 days after the administration of cisplatin (Heuberger *et al*, 1998). However, even in the presence of carnitine deficiency, patients may restore their carnitine pool by food intake or endogen production, and urinary loss may not be sufficient to cause a symptomatic deficiency. Finally, this prospective trial was performed in a population of patients with homogenous good physical status, but the effect of LC supplementation was not compared to a control group in a randomised fashion.

On the basis of these considerations, present data on LC as an ergogenic aid after cancer chemotherapy are not compelling. However, clinical trials which investigate putative therapies against chemotherapy-induced fatigue are almost lacking (Portenoy and Itri, 1999), and despite its limitations, the findings of this early study are intriguing and open new perspectives for future clinical trials. Further analyses are required to clarify the potential role of LC and we are planning to verify the efficacy of this compound in a randomised, placebo-controlled study.
